# Walking Modulates Active Auditory Sensing

**DOI:** 10.1523/JNEUROSCI.0489-25.2025

**Published:** 2025-09-29

**Authors:** Xinyu Chen, Liyu Cao, Roy Eric Wieske, Juan Prada, Klaus Gramann, Barbara F. Haendel

**Affiliations:** ^1^Institute of Psychology III, University of Würzburg, Würzburg 97070, Germany; ^2^Department of Neurology, University Hospital Würzburg, Würzburg 97080, Germany; ^3^Department of Psychology and Behavioural Sciences, Zhejiang University, Hangzhou 310058, China; ^4^The State Key Lab of Brain-Machine Intelligence, Zhejiang University, Hangzhou 311113, China; ^5^Department of Biopsychology and Neuroergonomics, Technical University of Berlin, Berlin 10623, Germany; ^6^Department of Bioinformatics, University of Würzburg, Würzburg 97074, Germany

**Keywords:** active sensing, alpha oscillation, auditory steady-state response, mobile brain imaging, natural walking, sensory processing

## Abstract

Walking provides the motor foundation for navigation, while navigation ensures that walking is purposeful and adaptive to environmental contexts. Sensory processing of environmental information acts as the informational bridge that connects walking and adaptive navigation. In the current study, we assessed if walking and the walking direction influences neuronal dynamics underlying environmental information processing. To this end, we conducted two experiments with 12 male and 18 female participants while they walked along an 8-shaped path. Auditory entrainment stimuli were continuously presented, and mobile electroencephalogram was recorded. We found increased auditory entrainment (auditory steady-state response) and early auditory evoked responses during walking compared with standing or stepping in place. We also replicated the well-established reduction of occipital alpha power during walking. The increase of auditory entrainment and the decrease of alpha power were correlated across participants. In the second experiment, randomly presented transient burst sounds led to a perturbation of the auditory entrainment response. The perturbation response was stronger during walking compared with standing; however, only when the burst sounds were presented to one ear but not to both ears. Most importantly, we found that the auditory entrainment was systematically modulated dependent on the walking path. The entrainment responses changed as a function of the turning direction. In general, the current work shows that walking changes auditory processing in a walking path-dependent way which might serve to optimize navigation. The walking path-related modulation might further reflect a shift of attention, marking a form of higher-order active sensing.

## Significance Statement

In this mobile electroencephalogram walking study, we uncovered a dynamic shift in auditory attention that aligns with changes in walking trajectory. Specifically, during turns, the brain prioritizes auditory input from the side of turn direction before the turn apex and then shifts preference to the opposite side. These findings reveal an active sensing mechanism that goes beyond simple motor adjustments to adjust sensory input but suggests that the brain dynamically optimizes the processing of sensory input, e.g., to facilitate navigation. This study offers potential applications for understanding spatial awareness in real-world environments and improving navigational aids.

## Introduction

The ability to move is an essential feature of living organisms. With the development of mobile electroencephalogram (EEG), growing evidence in human studies points to a close relation between body movements and cognition (Borghi and Cimatti, 2010; Koziol et al., 2012; Schmidt-Kassow and Kaiser, 2023; Stangl et al., 2023). For example, walking has been shown to influence a wide range of cognitive processes, from basic sensory processing (Yagi et al., 1999; Bullock et al., 2015; Cortney Bradford et al., 2019; Davidson et al., 2024) to higher-order processes like learning and creativity (Wilson and Gibbs, 2007; Frith et al., 2019; Lhuillier et al., 2021; Händel et al., 2024).

In electrophysiology, a robust finding in actively moving humans is the reduction of ongoing alpha power in the parieto-occipital cortex, which has been shown to be independent of electrode impedance, eye movements, and visual input (Ehinger et al., 2014; Lin et al., 2014; Zink et al., 2016; Kuziek et al., 2018; Shaw et al., 2018; Scanlon et al., 2019; Wagner et al., 2019; Cao et al., 2020; Robles et al., 2021). Alpha activity has been associated with neural inhibition (Klimesch et al., 2007; Jensen and Mazaheri, 2010). Consistent with this, stronger event-related potentials (ERPs) during body movements in the visual (Bullock et al., 2015; Dodwell et al., 2021; Chen et al., 2022a,b) and auditory (Scanlon et al., 2019; Vaughn et al., 2021) domains have been reported, which likely resulted from a disinhibition process due to alpha reduction (Chen et al., 2022a). Similarly, animal studies also showed that the firing rates of neurons in the primary visual cortex are modulated by the running speed and that this modulation persists in complete darkness (Niell and Stryker, 2010; Ayaz et al., 2013; Dipoppa et al., 2018; Neske et al., 2019; Kanamori and Mrsic-Flogel, 2022).

Locomotion can lead to a spatially specific change in sensory processing. Cao and Händel (2019) found that surrounding contrast impaired the detection of central targets during walking. This was accompanied by reduced occipital alpha power, suggesting less inhibition and enhanced visual processing for peripheral input [see also Benjamin et al. (2018) ]. Using a single-stimulus visual detection paradigm, Reiser et al. (2022) observed that posterior lateralized ERP latencies were shorter during walking than during stationary periods but only for stimuli presented in extrafoveal (peripheral) locations. These findings point to a redistribution of visual attention during natural movement as an optimization strategy for processing task-relevant input, which was supported by studies on head and eye movements while walking (Imai et al., 2001; Tuhkanen et al., 2021).

In the present study, we were interested in the effect of walking on auditory processing. It has been shown that walking involves cross-model activations (Brungart et al., 2019). Comparable brain responses to environmental boundaries in both auditory and visual maze navigation tasks have also been reported (Miyakoshi et al., 2021). Therefore, like in the visual domain, walking may influence auditory processing. In two experiments combining mobile EEG recording and a well-defined walking task, we showed an enhanced auditory entrainment (using the auditory steady-state response, ASSR) during walking compared with standing and stepping in place, which was robustly predicted by a decreased occipital alpha power during walking. Furthermore, we found that dichotic, but not diotic, tones led to an enhancement of ASSR perturbation during walking, which indicates a prioritized processing of auditory input with a peripheral origin in the environment. Importantly, when participants made turns during walking, dynamic changes in the auditory entrainment introduced by the input from the side of the turning direction were observed, indicating a change of auditory attention in accordance with the walking trajectory.

## Materials and Methods

### Participants

Thirty-five participants recruited from a local participants pool via the SONA system took part in the study (21 females; mean age = 28.05 years; SD = 3.51). Thirty-four participants completed both Experiment 1 and Experiment 2. One participant withdrew from the study shortly after Experiment 2 began; therefore only data from Experiment 1 was collected for this participant. All participants reported having normal bilateral hearing. They gave their written informed consent prior to the participation and received a payment of 10 euros per hour for their participation. The study was approved by the local ethics committee and complied with the European data protection law (DSGVO).

### Experimental design

The study included two experiments. The first experiment focused on the ASSR, and the second experiment aimed to also investigate a stimulus induced perturbation of the ASSR power. Both experiments were performed in a spacious room measuring ∼5 * 6 m. All auditory stimuli were generated using the PsychPortAudio software on a Dell laptop (model, Latitude E7440) and were presented through in-ear earphones (AirPods, Model A1602).

#### Experiment 1

**Figure 1.**
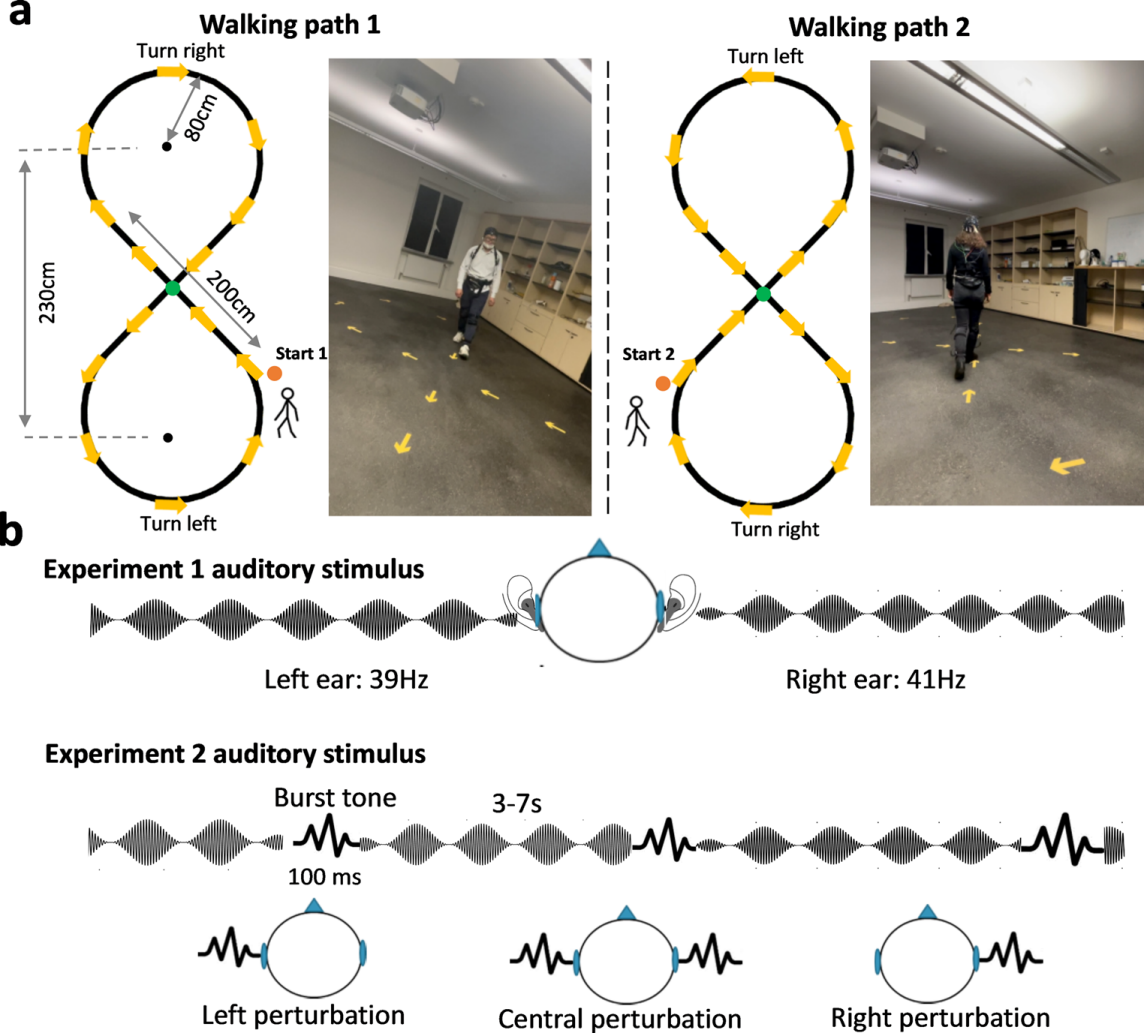
The experimental setup. ***a***, Movement task, In Experiment 1, there were three testing conditions: stepping, standing, and walking. In Experiment 2, there were two movement conditions: standing and walking. In the standing condition, participants were instructed to stand in the center of the marked walking pathway (indicated by a green dot). In the stepping condition, participants were asked to walk on the spot in the same location as in the standing condition. In the walking condition, participants walked in an “8”-shaped walking path. The start location is marked with an orange dot. Half of the participants walked on Path 1, starting with a rightward turn and then a leftward turn. For the other half of the participants, the order of turn direction was reversed (Path 2). The two walking paths were indicated by yellow arrows attached to the ground (the black “8” path in the figure is for illustration purposes only and was not presented in the actual experiment). ***b***, The auditory stimuli, In experiment 1, participants listened to tones that were modulated at frequencies of 39 and 41 Hz for the left and right ear, respectively. In Experiment 2, in addition to the continuously played tones, intermittent burst sounds lasting 100 ms were presented. Bursts could occur simultaneously in both the 39 and 41 Hz tones, and participants reported hearing the bursts in a midline location; hence we termed it central burst. The burst could also occur only in the left ear, i.e., the 39 Hz tone was interrupted, or in the right ear, i.e., the 41 Hz tone was interrupted. The interruption was then perceived either in the left or right periphery. Participants were not instructed to respond to or attend to the tones in any particular way.

Each participant completed two testing blocks, each containing a stepping, a walking, and a standing condition. Half of the participants completed the three conditions with an order of stepping–walking–standing, and the other half of the participants with an order of standing–walking–stepping. In the standing condition, participants were instructed to stand in the center of the marked walking pathway (Fig. 1*a*, green dot). In the stepping condition, participants were asked to walk on the spot in the same location as in the standing condition (Fig. 1*a*, green dot). In the walking condition, participants were asked to walk in an “8”-shaped pathway (Fig. 1*a*, left panel). Specifically, the walking path included walking straight (heading to left/right, indicated by dashed yellow arrows), making a semicircular left turn, and making a semicircular right turn. The walking path was indicated by arrows attached to the ground, but participants were asked not to strictly fixate at the exact walking path (the black 8 path in the figure is for illustration purposes only and was not presented in the actual experiment). Note that the starting location (Fig. 1*a*, orange dots) for walking was balanced among participants to control for external variables, such as the location of the light source and the visual input provided by the arrow. Figure 1*a* Walking Path 1 illustrates the experimental conditions for participants who first started by walking straight to the left and then turned right. The other half of the participants began by walking straight to the right and then turned left, and accordingly, all yellow arrows were also flipped to align with this condition's walking instruction, as indicated by Figure 1*a* Walking Path 2. The walking speed was trained before the start of the experiment to ensure that participants took ∼12 s to complete one full “8”-shaped pathway. The experimenter also visually monitored the speed throughout the testing sessions. The testing duration of stepping, walking, and standing was 120, 480, and 120 s, respectively. The total testing time for each block was ∼12 min.

During the experiment, participants were exposed to a continuous stream of sinusoidal amplitude-modulated tones to elicit an ASSR. The tone in the left ear was modulated at 39 Hz and at 41 Hz in the right ear (Fig. 1*b*). We chose these two frequencies as ASSR power was found to reach a maximum response ∼40 Hz in humans (Galambos et al., 1981; van der Reijden et al., 2001). The carrier frequency was set to 1,000 Hz, with a modulation depth of 100%. The auditory stimuli had a sampling rate of 44,100 Hz. Participants were not required to perform any behavioral task in response to the tones.

#### Experiment 2

**Figure 2.**
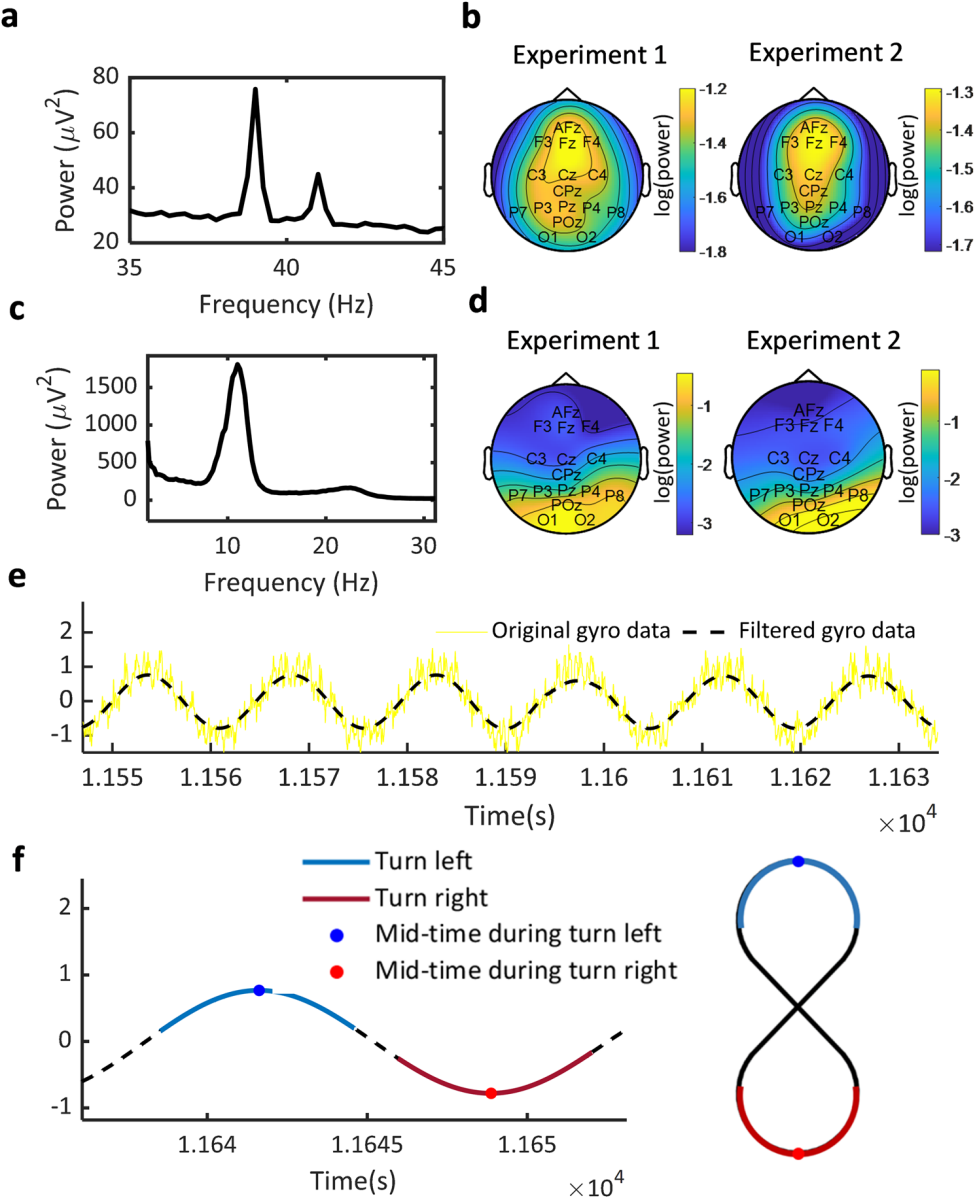
Data preprocessing. ***a***, Referenced and filtered EEG data were subjected to ICA. The ICA component with the highest ASSR was selected for further analysis. An example component is shown. ***b***, The topography of all selected components’ ASSR power response for all participants are shown for Experiment 1 and Experiment 2. ***c***, ***d***, Example alpha component and the topography of alpha power response for Experiment 1 and Experiment 2. ***e***, Example period of raw (black line) and low-pass filtered gyroscope data (blue line). ***f***, The periods corresponding to turning left (blue areas) and turning right (red areas) are extracted based on the low-pass filtered gyroscope data. The peak of the sine wave, representing the middle time point during the actual turning path, was defined as the 0 time point (marked with a blue and red dot, respectively) and used for the later analysis of ASSR lateralization dependent on the turning direction.

Each participant completed four testing blocks, each containing a walking and a standing condition similar to Experiment 1. Half of the participants began with the standing condition, while the other half began with the walking condition. The starting point (Fig. 1*a*, 1 or 2) of walking was again balanced among participants to control for potential external confounding variables. The total testing duration for the walking and standing condition was ∼8 and 2 min in each block, respectively.

Participants were exposed to a continuous stream of sinusoidal tones, which were amplitude-modulated at 39 Hz (left ear) and 41 Hz (right ear) as in Experiment 1. To perturb the auditory stimuli, burst sounds (white noise) lasting 100 ms were intermittently presented diotically or dichotically at unpredictable latencies and order (Fig. 1*b*). For diotic perturbation (central burst), the burst sound was presented to both ears simultaneously, and almost all participants reported hearing a burst sound in the frontal midline when they were asked by the experimenter regarding their perception of the diotic perturbation. For dichotic perturbation (left or right burst), the burst sound was only presented in the left ear or the right ear. Participants in this case reported hearing a burst sound in a left or right peripheral location, respectively. In the standing block, participants experienced eight bursts at intervals of 3–7 s for each burst type (central, left, or right). In the walking block, participants experienced 32 bursts also every 3–7 s for each burst type (central, left, or right) to ensure that enough trials with bursts could be recorded during turning left and right. As in Experiment 1, participants were not instructed to respond to or attend to the tones in any particular way.

### Data acquisition

EEG data were recorded using the Smarting mobile EEG system (mBrainTrain) with 24 bits resolution. The EEG system provides 24 channels and used wet electrodes located according to the 10% system (covering frontal central, parietal, temporal, and occipital areas) with a sampling rate of 500 Hz. Among the 24 electrodes, 18 electrodes were used for EEG recording (with one electrode on each earlobe for possible rereferencing). Another six electrodes, which were placed around the eyes (three electrodes for each eye: one above and one below the eyes, one near the outer canthus), were used for electrooculogram (EOG) recording. The EEG signal amplifier and data transmitter have wireless data transmission via Bluetooth. The electrode impedance was kept below 15 kOhm.

Motion data were recorded (velocity and acceleration; sampling rate, 120 Hz) with a Perception Neuron system (https://neuronmocap.com/products/perception_neuron; Noitom). Three-dimension velocity and three-dimension acceleration data were collected from three sensors: one attached to each ankle (a few centimeters above the lateral malleolus) and the third one attached to the participant's back (at the waist level). Data transmission was also achieved via Bluetooth.

The software Lab Streaming Layer (https://github.com/sccn/labstreaminglayer) was used to collect and synchronize all data streams (event markers, EEG, and motion data; Kothe et al., 2024). Stimulus generation and presentation were coded in MATLAB (The Mathworks, R2019b) using the Psychtoolbox (Kleiner et al., 2007).

### Data analysis

#### Preprocessing

The EEG data analysis was performed using the Fieldtrip toolbox (Oostenveld et al., 2011) and custom scripts developed in MATLAB (The MathWorks). For both experiments, we excluded two participants' data due to data transmission errors of the motion data. Additional three datasets recorded in Experiment 1 and four datasets recorded in Experiment 2 were excluded because of the failure to elicit an ASSR with a clear peak power at either 39 or 41 Hz. After these exclusions, data from 30 participants were included in the final analysis of Experiment 1, and 28 participants were included in the final analysis of Experiment 2.

EEG data from each participant were first re-referenced to the average of two earlobe electrodes. The data were then high-pass filtered at 1 Hz and low-pass filtered at 100 Hz. A band-stop filter between 49.5 and 50.5 Hz was applied to remove 50 Hz line noise. The data were then reduced to 16 dimensions using principal component analysis, followed by independent component analysis (ICA) using the “runica” (Infomax) approach (Delorme and Makeig, 2004). The power spectrum of each ICA component was obtained using Welch's method with a 1 s time window and 50% overlap.

The components with clear ASSR signal at 39 or 41 Hz were selected for further analysis. An example component can be seen in Figure 2*a*. Note that we did not have specific requirements regarding the topographic distribution when selecting the ASSR components. On average, 1.67 (SD = 0.66) ASSR components were obtained per participant in Experiment 1 and 1.89 (SD = 0.88) ASSR components were obtained per participant in Experiment 2. The average topography of the components’ ASSR power from all participants can be seen in Figure 2*b*. In general, the frontocentral area showed the largest ASSR power response. For the selection of the alpha components, the criteria from our previous study were used (Cao et al., 2020). Specifically, four requirements should be met: (1) There should be a local power peak between 6 and 14 Hz in the component's power spectrum; (2) the width of the local peak, which was defined as the frequency width between the two adjacent local minimums, was required to be at least 4 Hz (avoiding noisy transient peaks). If there was more than one local peak, the local peak with the largest width was taken (also for Step 3). (3) The power at the local peak frequency had to be at least three times higher than the mean power between 20 and 50 Hz (avoiding components with a broadband high power); (4) in the ICA topography, the maximum absolute weight from sensors in the occipital area (O1, O2, and POz) had to be larger than the maximum absolute weight from all other sensors. Following these selection criteria, eight participants in Experiment 1 and five participants in Experiment 2 were excluded as no components fulfilled the above requirements. On average, with participants who fulfilled the criteria (remaining participants in Experiment 1, 22; Experiment 2, 23), 1.47 (SD = 1.07) alpha components were obtained per participant in Experiment 1 and 1.83 (SD = 0.78) components were obtained per participant in Experiment 2. An example alpha component can be seen in Figure 2*c*. The topography of the alpha power response for all participants can be seen in Figure 2*d* for the two experiments. The occipital electrodes showed the strongest alpha power response. Both ASSR and alpha components were subsequently back-projected to the sensor space for further analysis.

#### The walking path extraction (both Experiments 1 and 2)

The walking path was extracted in both Experiment 1 and Experiment 2, using the same procedure based on the gyroscope data recorded during walking. For each left and turn right, the 0 time point was defined as the peak in the low-pass filtered gyroscope data (Fig. 2*e*). This corresponds to the middle time point during the turn (Fig. 2*f*, marked with a blue dot for left turn and red dot for right turn). The average number of left turns for each participant was 33.92 (SD = 6.61), and the number of right turns was 37.05 (SD = 5.52). The duration of left turns was on average 6.41 s (SD = 0.97), and the duration of right turns was on average 6.43 s (SD = 0.97). A turn trial was then defined as the time window of 0 time point ± 2 s, resulting in a 4 s trial.

#### The ASSR and alpha power analyses (both Experiments 1 and 2)

For both experiments, the frontocentral electrodes (AFz, Fz, F3, F4, Cz) were included in the ASSR-related analyses, as they covered the main response region where the ASSR power was strongest. We first compared the overall ASSR power in Experiment 1 with the three movement states (standing vs walking vs stepping). To this end, for each movement state, we cut the preprocessed EEG data based on the identified ASSR components into 1 s periods with a 50% overlap in time. In order to minimize the influence of aperiodic noise on the ASSR power, we parameterized the aperiodic component using the FOOOF tool as implemented in FieldTrip (Donoghue et al., 2020). This tool can separate the aperiodic 1/*f* activity from the periodic activity (defined as power over and above broadband 1/*f* activity) of the power spectrum. This method has been applied in many mobile EEG studies (Canessa et al., 2020; Protzak and Gramann, 2021). Individual power values were parameterized as the power of the identified peaks between 1 and 48 Hz. With the parameterized power, a two-way (movement state, step vs stand vs walk; ASSR frequency, 39 vs 41 Hz) repeated-measures ANOVA was performed with the parameterized ASSR power. The comparison in Experiment 2 focused on the walking and standing conditions. Again, a two-way (movement state, stand vs walk; ASSR frequency, 39 vs 41 Hz) repeated-measures ANOVA was performed with the parameterized ASSR power.

Furthermore, we examined whether walking led to a decrease in alpha power, as reported in previous studies. The parameterized power was obtained using the same method as for the ASSR power but was based on the identified alpha components. In Experiment 1, a one-way (movement state, step vs stand vs walk) repeated-measures ANOVA was performed with the alpha power (8–14 Hz) averaged over the two lateral occipital electrodes (O1 and O2). In Experiment 2, we compared the average alpha power using paired-sample *t* tests between stand and walk. Throughout the results in the manuscript, the alpha power and ASSR power always refer to the parameterized power.

#### The ASSR lateralization analyses

The time-resolved ASSR power was obtained through a Hilbert transformation of the bandpass (±0.5 Hz of the frequency of interest) filtered data in each block before epoching data into turning trials. An ASSR lateralization index was then computed which indexed the lateralization of entrainment at each time point.

ASSR lateralization index:

$$\frac{39\mathrm{Hz}\;\mathrm{ASSR}\;\mathrm{power}\;\text{–}\;41\mathrm{Hz}\;\mathrm{ASSR}\;\mathrm{power}}{39\mathrm{Hz}\;\mathrm{ASSR}\;\mathrm{power}+41\mathrm{Hz}\;\mathrm{ASSR}\;\mathrm{power}}.$$

Since the 39 Hz ASSR tone was consistently presented to the left ear, while the 41 Hz tone was consistently presented to the right ear, the ASSR power at 39 Hz would reflect the strength and synchrony of the neural activity in response to the left-side input, while the ASSR power at 41 Hz would reflect the strength and synchrony of the neural activity in response to the right-side input. Therefore, a larger ASSR modulation index indicates a relatively stronger processing of the input from the left ear, while a smaller ASSR modulation index indicates a relatively stronger processing of the input from the right ear.

To check whether the ASSR modulation index was influenced by the turning direction, a cluster-based permutation statistical method was used to assess if the ASSR lateralization index between the left turn and right turn was significantly different (Maris and Oostenveld, 2007). The data were shuffled (1,000 permutations) to estimate a “null” distribution of the *t* values based on cluster-level statistics (cluster-defining threshold, *p* < 0.05). Significance of the *t* value of the original test is assumed if the *t* value falls above the upper 5% of permeation *t* values. The time window of the statistical comparison was between ([−2 2] s).

#### The ASSR perturbation analyses (Experiment 2 only)

##### The modulation of ASSR perturbation by movement condition

To investigate how the sensory-specific processing was modulated by movement and burst location, we compared the power of the burst sound-evoked ASSR perturbation. A perturbation trial was time-locked to the burst sound onset (Time 0) and included 2 s data both before and after the burst sound onset, resulting in a 4 s trial.

The burst-tone-evoked ASSR perturbation was calculated after averaging over all trials in each condition. A baseline correction was performed using a prestimulus window ([−400 0] ms) with log transformation. The strength of the ASSR perturbation was calculated as the average power between ([0 700] ms) after the burst sound onset. A three-way (movement state, stand vs walk; burst location; left vs right vs central; ASSR frequency, 39 vs 41 Hz) repeated-measures ANOVA was performed with the mean ASSR power perturbation as a dependent measure. Throughout the manuscript, we used a Greenhouse–Geisser correction for ANOVA results when necessary, and statistical significance was defined as *p* < 0.05.

##### The modulation of ASSR perturbation by walking path direction

The onset of the burst sound was further classified based on the direction of turning during which it occurred, i.e., bursts that occurred while turning left and bursts that occurred while turning right. A three-way (turn direction, left vs right; perturbation location; left vs right vs central; ASSR frequency, 39 vs 41 Hz) repeated-measures ANOVA was performed with the mean ASSR power perturbation.

#### ERP analyses (Experiment 2 only)

To check the auditory evoked response induced by the burst sound, the filtered data were epoched into trials with time window of ±2 s around the burst sound. The EEG data were baseline-corrected by applying a 700 ms prestimulus (averaged over ([−700 0]) ms) absolute baseline to each trial. The grand average ERP was again based on the frontocentral electrodes (AFz, Fz, F3, F4, Cz). We compared the first and second positive components (named P1 and P2 component henceforth). The time windows of the P1 and P2 components were selected by using a 50 ms window centering the positive peaks of the grand average. In this ERP analysis, a two-way (movement state, stand vs walk; burst location, left vs right vs central) ANOVA was performed with the P1 and P2 component amplitude separately.

#### Correlational analyses

To investigate the potential correlation between the modulation of alpha power due to walking and the neural markers of sensory processing, correlational analyses were performed. Except for the exploratory test for the evoked P2 component, all correlations were one-sided and based on our hypotheses. We utilized the Spearman correlation coefficient for these analyses. Outlier rejection was conducted using the method from the Robust Correlation Toolbox (Pernet et al., 2013). Specifically, outliers were identified as the intersection of bivariate flags based on three robust methods: the boxplot method, the median absolute deviation method, and the S-outlier method. Only data points flagged by all three approaches were considered outliers.

Frequency analysis: first, correlation analysis was performed between the alpha power modulation and the ASSR power modulation during walking, averaging between the two frequencies (39 and 41 Hz). This aimed to assess the relationship between alpha power and ASSR power modulation due to walking. Additionally, we examined the relationship between the alpha power modulation and the ASSR power perturbation modulation due to walking, again averaging between the two frequencies (39 and 41 Hz).

ERP analysis: Following up on previous studies finding a relationship between alpha power and early evoked responses, we examined the relationship between the modulation of the early burst-tone-evoked response (P1) and modulation of the alpha power. Additionally, the correlation between the modulations of the P1 component amplitude and ASSR power was also calculated. The relationship between the modulations P2 component and ASSR perturbations was additionally assessed to determine whether the P2 component marks similar processes as those indicated by ASSR perturbations that was modulated by walking.

## Results

### Enhanced ASSR power was related to the decreased alpha power during walking

**Figure 3.**
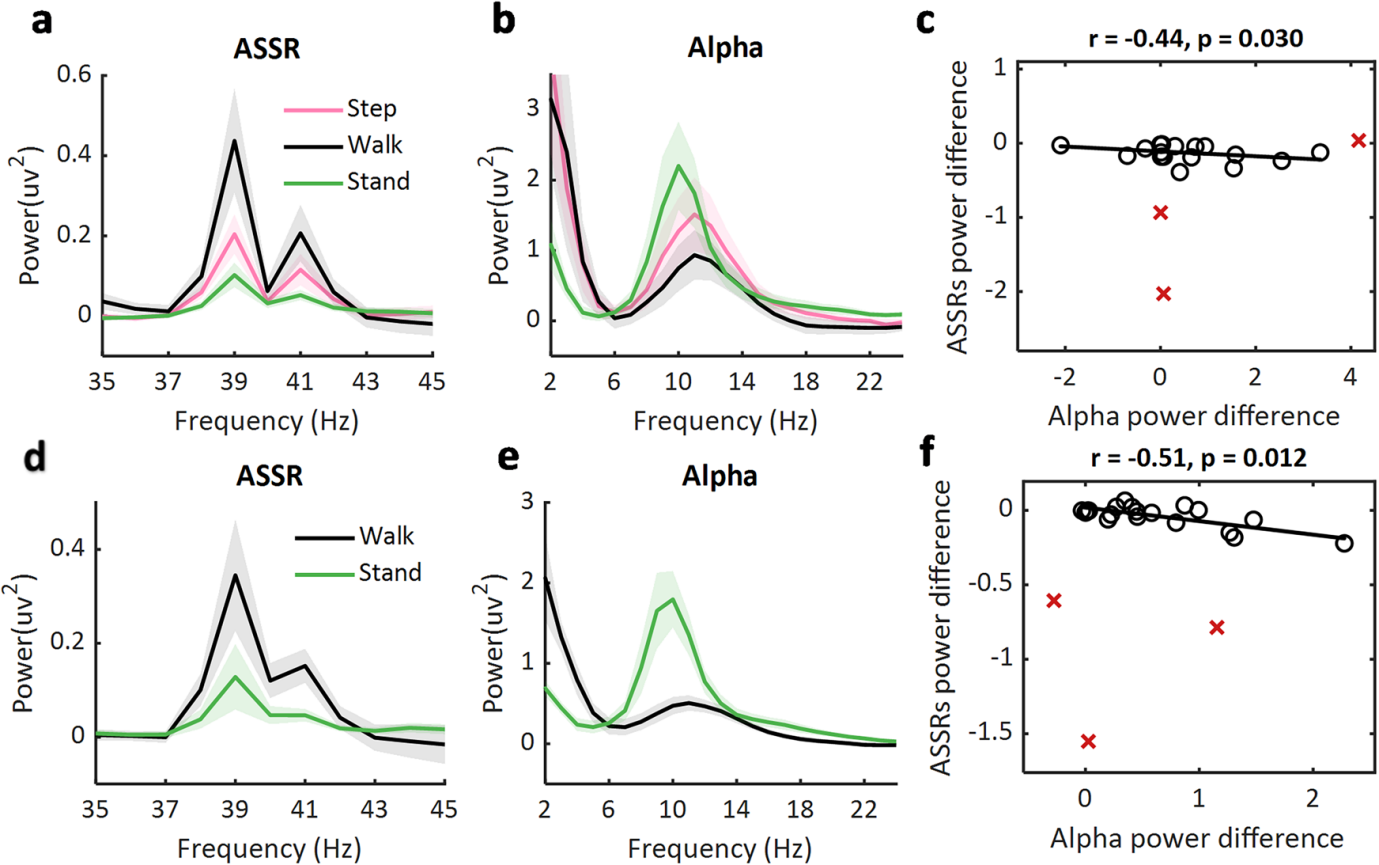
ASSR power and alpha power during different movement conditions. ***a***, The power responses between 35 and 45 Hz, averaged over frontocentral electrodes (AFz, Fz, F3, F4, Cz) in stepping (pink line), walking (black line), and standing (green line) conditions, are shown for Experiment 1. Both the 39 Hz ASSR power and 41 ASSR power were significantly higher during walking compared with stepping and were higher compared with standing. Shading lines indicate ±standard error. ***b***, The power averaged over the two occipital electrodes (O1 and O2) is shown between 3 and 25 Hz during stepping, standing, and walking for Experiment 1. The average alpha power (8–14 Hz) was significantly higher during standing compared with stepping and was the smallest during walking. ***c***, The ASSR power difference (averaged over 39 and 41 Hz) was negatively correlated with the alpha power difference (stand–walk). Three outliers (marked with red crosses) were excluded. ***d–f***, The same is shown for Experiment 2; accordingly, only standing and walking conditions were present. The results replicated the walking-induced modulation of ASSR power (***d***), alpha power (***e***), and a significant correlation between alpha power and ASSR power (***f***) as shown in Experiment 1.

We first compared the overall ASSR power between the three movement states in Experiment 1. The two-way (movement state, step vs stand vs walk; ASSR frequency, 39 vs 41 Hz) repeated-measures ANOVA was performed with the ASSR power averaged over frontocentral electrodes (AFz, Fz, F3, F4, Cz). The results showed a main effect of movement state (*F*_(2,58)_ = 7.80; *p* = 0.007), with the ASSR power during walking (*M* = 0.32; SD = 0.52) being higher than during stepping (*M* = 0.16; SD = 0.23; post hoc *t*_(29)_ = 2.74; *p* = 0.011) and during standing (*M* = 0.07; SD = 0.11; post hoc *t*_(29)_ = 2.86; *p* = 0.008; Fig. 3*a*). The ASSR power during stepping was also higher than during standing (post hoc *t*_(29)_ = 2.49; *p* = 0.019). The main effect of ASSR frequency was also significant (*F*_(1,29)_ = 5.00; *p* = 0.033), showing that the power for 39 Hz ASSR (*M* = 0.13; SD = 0.36) was higher than the power for 41 Hz (*M* = 0.24; SD = 0.22). No other effect was statistically significant.

The one-way (movement state, step vs stand vs walk) repeated-measures ANOVA performed with the alpha power averaged over the two lateral occipital electrodes (O1 and O2) revealed a significant main effect of movement state (*F*_(2,42)_ = 4.49; *p* = 0.033). The post hoc *t* tests showed that the alpha power was higher during standing than during walking (*t*_(21)_ = 2.64; *p* = 0.015). The alpha power during stepping was also significantly higher than during walking (*t*_(21)_ = 2.85; *p* = 0.010). The difference between standing and stepping did not reach significance (*t*_(21)_ = 1.23; *p* = 0.232; Fig. 3*b*).

To test whether the ASSR power enhancement was associated with the deceased occipital alpha power during walking, we performed a between-participant correlation between the alpha power difference (stand–walk) and ASSR power difference (stand–walk). A significant negative correlation was found (*r* = −0.44; *p* = 0.030; one-tailed; Fig. 3*c*), showing that lower alpha power was associated with higher ASSR power.

**Figure 4.**
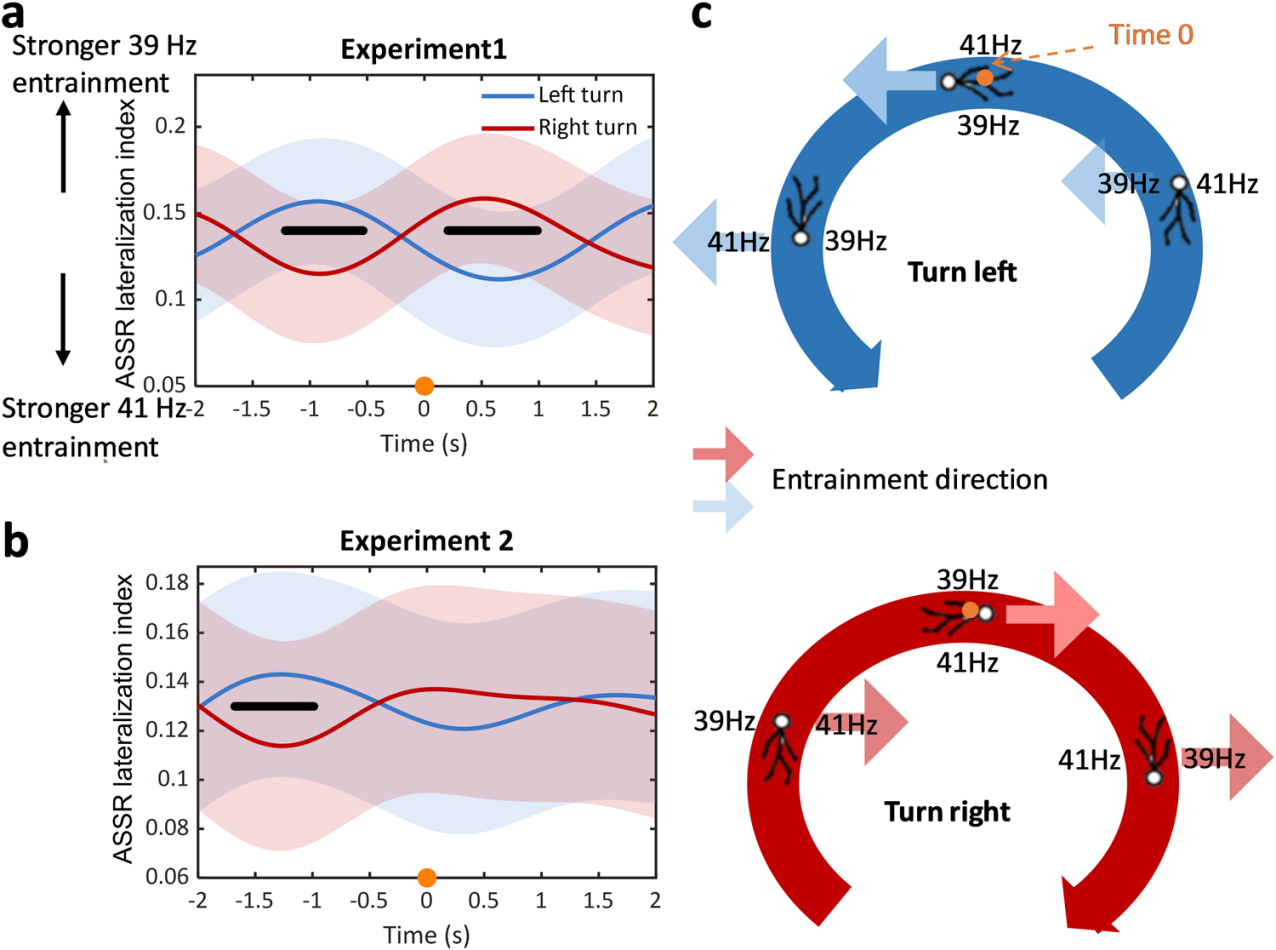
The ASSR lateralization index is modulated by the walking path. ***a, b***, The ASSR lateralization index showed a different pattern dependent if the subjects executed a left turn (blue lines) or a right turn (red line). Before Time Point 0 (marked with an orange dot in the schematic walking path in ***c***), the ASSR lateralization was significantly more positive for the left turn in both Experiment 1 and Experiment 2. This indicates that the power of the 39 Hz ASSR (presented to the left ear) was stronger than the 41 Hz power (presented to the right ear). In the time window 500 ms before Time Point 0, the 39 and 41 Hz power was similar. After Time Point 0, the ASSR lateralization was significantly more positive for the right turn, indicating that the 41 Hz ASSR power was now stronger than during the left turn. The shaded area indicates ±standard error. The black thick line marks the time windows of significance (FDR adjustment for multiple comparisons). ***c***, The walking direction and the position of the left (39 Hz) and right (41 Hz) ear entrainment within a turn are indicated by the stick figure and the frequency labels. The arrows indicate the side of stronger entrainment as shown in ***a*** and ***b***.

We compared again the ASSR power between the two movement states in Experiment 2. The two-way (movement state, stand vs walk; ASSR frequency, 39 vs 41 Hz) repeated-measures ANOVA was performed with the ASSR power averaged over the frontocentral electrodes (AFz, Fz, F3, F4,Cz). The results showed a main effect of movement (*F*_(1,27)_ = 20.67; *p* < 0.001), with the ASSR power during walking (*M* = 0.23; SD = 0.31) being higher than during standing (*M* = 0.08; SD = 0.16; post hoc *t*_(27)_ = −4.55; *p* < 0.001; Fig. 3*a*). We then performed *t* tests comparing the alpha power between standing and walking. The results showed that alpha power was smaller during walking compared with standing (*t*_(24)_ = 4.53; *p* < 0.001; Fig. 4*e*). Moreover, the correlation between the modulation of ASSR power and alpha power was also significant (*r* = −0.53; *p* = 0.009; one-tailed; Fig. 4*f*).

### The strength of ASSR lateralization is modulated by the walking path

#### Experiment 1

The average ASSR lateralization index for the left and right turn was computed to reflect the difference in the entrainment response between left and right ears. A larger ASSR lateralization index indicated more attention to the left ear (or left spatial field). The ASSR lateralization was time-locked to the midturn time point, which was the time when participants were at the apices of the 8-shaped walking path. A cluster-based permutation *t* test between left turns and right turns revealed a significant difference between the two turn directions during the time window before the midturn time point ([−1.20 −0.53] s; *p* = 0.016) as well as after the midturn time point ([0.20 0.99]s; *p* = 0.028; Fig. 4*a*). The pattern indicated that spatial auditory processing changed dynamically in accordance with the turning behavior. Processing was stronger at the side of the turn (e.g., a left turn, increased processing of left spatial input) in the starting phase of turning (before the midturn time point at 0 s). However, at the later phase of turning, after the midturn, processing changed to be stronger at the opposite side of the turn (e.g., a left turn, increased processing of the right spatial input).

#### Experiment 2

Experiment 1 was designed and optimized for the ASSR lateralization analysis, whereas Experiment 2 was designed to analyze the processing of a perturbing stimulus, in our case a sound burst. Nevertheless, we tested whether the modulation of ASSR lateralization by the walking path can be observed in Experiment 2 despite the presence of perturbing stimuli. Indeed, the ASSR modulation index showed a similar pattern (Fig. 4*c*) as in Experiment 1. The cluster-based permutation *t* test between left turn and right turn revealed a significant difference between left turn and right turn during the time window before the midturn time point ([−1.68 −0.99] s; *p* = 0.006). However, the difference between left turn and right turn did not reach significance after the midturn time point.

To rule out potential confounds from muscle-related broad-frequency activity, we tested whether the observed lateralization pattern was specific to the ASSR frequencies. We computed the lateralization index using neighboring frequencies (37 and 44 Hz) and applied the same cluster-based permutation test as in the original analysis. No systematic modulation emerged between left-turn and right-turn conditions for Experiment 1 and Experiment 2 (Text S1; Fig. S1), and no significant differences in lateralization index were detected. Additionally, the horizontal EOG (HEOG) signal time-locked to the turning events were analyzed. There was no consistent or directionally specific drift in the HEOG signal that could account for the observed ASSR modulation in both Experiment 1 and Experiment 2 (Text S2; Fig. S2).

### Peripheral ASSR power perturbation modulation was enhanced during walking

**Figure 5.**
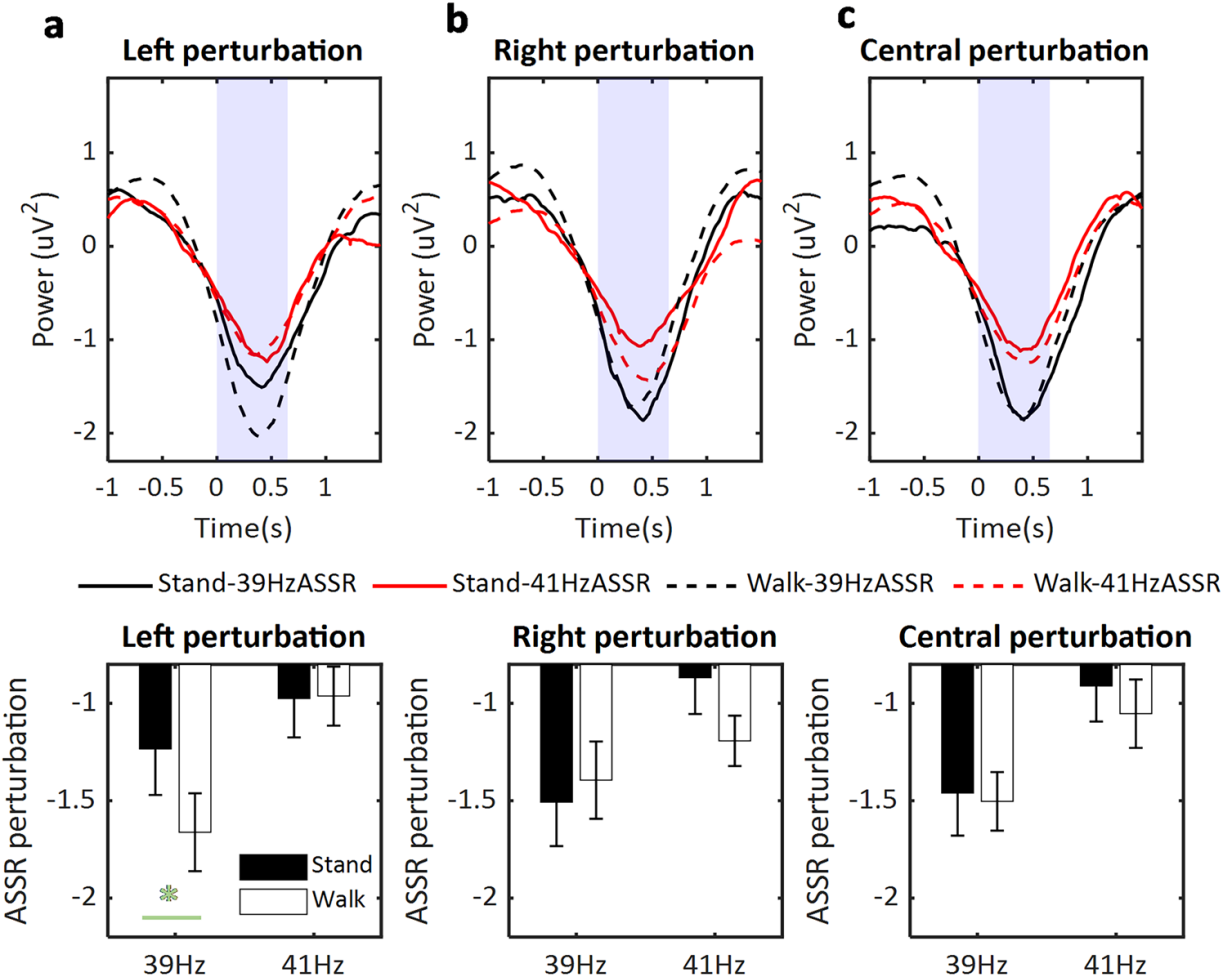
ASSR power perturbation induced by burst sounds during walking compared with standing. ***a***, The upper panel shows the ASSR power perturbation (introduced by a left burst sound starting at time point 0 s) in standing (solid line) and walking (dashed line) conditions. The lower panel shows the results of statistical comparison. The ASSR power ([0 0.7] s, marked with purple) for a left burst sound showed a significantly stronger change during walking compared with standing for the 39 Hz ASSR power perturbation (left-side ASSR) but not for the 41 Hz ASSR power perturbation (right-side ASSR). ***b***, ***c***, Same as ***a*** but for the right burst sound or left and right simultaneous (central) burst sound, respectively. When the burst sound appeared on the right side, only the 41 Hz ASSR power perturbation was more strongly affected by the tone during walking compared with standing. When both 39 and 41 Hz tones were interrupted by a burst sound, no significant difference was observed between standing and walking. The green asterisks (*) indicate *p* < 0.05.

To test if dichotic sensory input would more strongly affect the ASSR signal during walking, a three-way (movement state, stand vs walk; burst location, left vs right vs central; ASSR frequency, 39 vs 41 Hz) repeated-measures ANOVA was performed. As the dependent variable, we used the mean amplitude the ASSR perturbation response (39 and 41 Hz, respectively) in the frontocentral electrodes (AFz, Fz, F3, F4, Cz) during ([0 700] ms) after the burst sound onset. Results showed a significant interaction between movement state, perturbation location, and ASSR frequency (*F*_(2,54)_ = 3.36; *p* = 0.049). Furthermore, post hoc *t* tests indicated that when the burst sound appeared in the left ear (perturbing the 39 Hz tone), the 39 Hz ASSR power perturbation was more strongly modulated during walking (*M* = −1.66; SD = 1.06) compared with standing (*M* = −1.23; SD = 1.25; *t*_(27)_ = 2.08; *p* = 0.047; Fig. 5*a*). When the burst sound appeared in the right ear (perturbing the 41 Hz tone), the 41 Hz ASSR power perturbation was more strongly modulated during walking (*M* = −1.19; SD = 0.68) compared with standing (*M* = −0.87; SD = 1.05); however the post hoc *t* test was marginally significant (*t*_(27)_ = 1.92; *p* = 0.065; Fig. 5*b*). When both 39 and 41 Hz tones were interrupted by a burst (perception of a central tone), no significant difference was observed between standing and walking in either 39 Hz (*t*_(27)_ = 0.22; *p* = 0.826) or 41 Hz ASSR perturbation response (*t*_(27)_ = 0.78; *p* = 0.442; Fig. 5*c*). Additionally, a significant main effect of ASSR frequency was observed (*F*_(1,27)_ = 14.30; *p* < 0.001), indicating that the 39 Hz ASSR perturbation response (*M* = −1.46; SD = 0.93) was stronger than the 41 Hz ASSR perturbation response (*M* = −0.99; SD = 0.71). No other significant effects were found.

We also investigated whether the difference in ASSR perturbation response between standing and walking (average over perturbation locations) could be predicted by the movement-related difference in ongoing alpha power. However, the correlation analysis yielded a nonsignificant result (*r* = −0.18; *p* = 0.386), indicating that the modulation of ASSR perturbation during free walking was not associated with the modulation of ongoing alpha activity during walking.

### Modulation of peripheral ASSR perturbation was not dependent on the walking path

**Figure 6.**
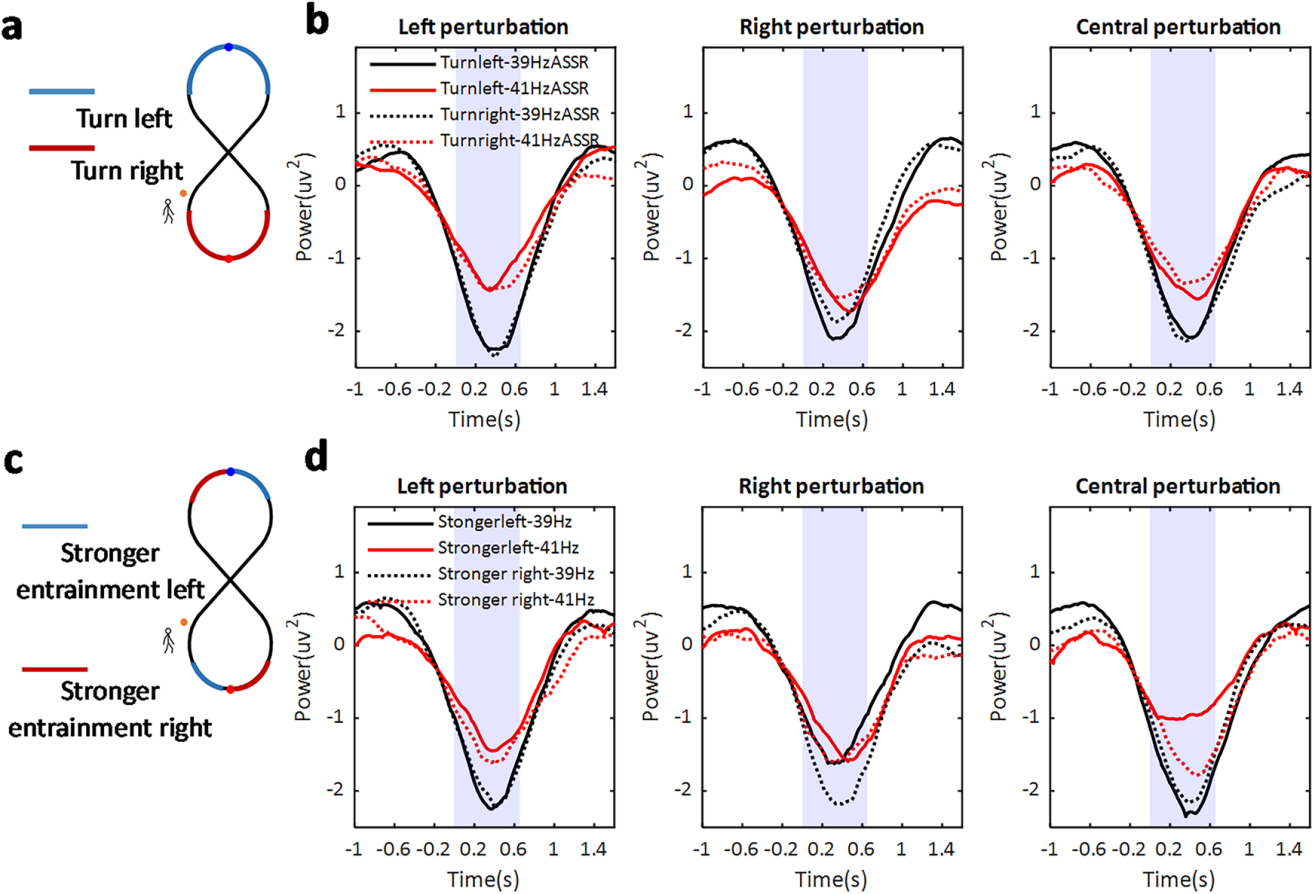
The modulation of peripheral ASSR perturbation during walking. ***a***, The perturbation grouping (solid and broken lines) as shown in ***b*** was based on a [±2 s] time window around the time point of local maximum/minimum (marked with a dot) during the left turn (blue) and right turn (red). ***b***, The 39 Hz power perturbation (plotted in black) and 41 Hz power perturbation (plotted in red) are shown separately if happened during the left turn (solid lines) and right turn (dashed lines), for the left burst sound (left panel), right burst sound (central panel), and central burst sound (right panel) conditions. The power perturbation, average over the time window between ([0 0.7] s), was statistically compared (marked in purple). ***c***, The perturbation grouping (solid and broken lines) as shown in ***d*** was based on a [2 s] time window before (blue) and after (red) the time point of local maximum/minimum (marked with a dot) during the left and right turn. ***d***, Same as ***b*** but grouping was based on the time periods marked in ***c***.

In the next step, we investigated whether the strength of the ASSR perturbation at peripheral location depended on the walking path by testing if the strength was different between turn directions. First, the perturbation trials were grouped based on the perturbation side (left side, 39 Hz; right side, 41 Hz) and whether the perturbation happened during a left or a right turn (Fig. 6*a*, the left-turn path is marked in blue; the right-turn path is marked in red). A three-way (turn direction, left vs right; perturbation location, left vs right vs central; ASSR frequency, 39 vs 41 Hz) repeated-measures ANOVA was performed with the average ASSR power perturbation between the frontocentral electrodes (AFz, Fz, F3, F4, Cz) within 700 ms after the burst sound onset. In addition to the main effect of ASSR frequency (*F*_(1,27)_ = 8.84; *p* = 0.006), only the interaction between the perturbation location and the ASSR frequency was significant (*F*_(2,54)_ = 4.51; *p* = 0.017): the 39 Hz ASSR power was most strongly modulated in the left perturbation condition, while the 41 Hz ASSR power was most strongly modulated in the right perturbation condition. No other effect was found to be statistically significant, including the interaction between turn direction, ASSR perturbation, and ASSR frequency (*F*_(2,54)_ = 0.99; *p* = 0.375; Fig. 6*b*).

However, as we saw that the ASSR was modulated by the turn direction in such a way that the lateralization flipped around Time Point 0, thereby always preferring the absolute direction of the run, we also tested the perturbation strength accordingly. The perturbation trials were therefore grouped based on whether they happened in the time during which the left ear input (39 Hz) or the right ear input (41 Hz) was preferred (Fig. 6*c*, stronger left entrainment time was marked in blue; stronger right entrainment was marked in red). This three-way (stronger entrainment direction, left vs right; perturbation location; left vs right vs central; ASSR frequency, 39 vs 41 Hz) repeated-measures ANOVA with the perturbation power within the first 700 ms showed a significant main effect of ASSR frequency (*F*_(1,27)_ = 6.18; *p* = 0.019). This suggests that the 39 Hz ASSR perturbation amplitude was more strongly modulated compared with 41 Hz ASSR power (Fig. 6*d*). No other effects were statistically significant.

### ERP locked to the burst tone onset

The ERP was time-locked to burst onset and analyzed again at frontocentral electrodes (AFz, Fz, F3, F4, Cz), specifically focusing on the P1 component ([140 190] ms) and the P2 component ([300 350] ms).

**Figure 7.**
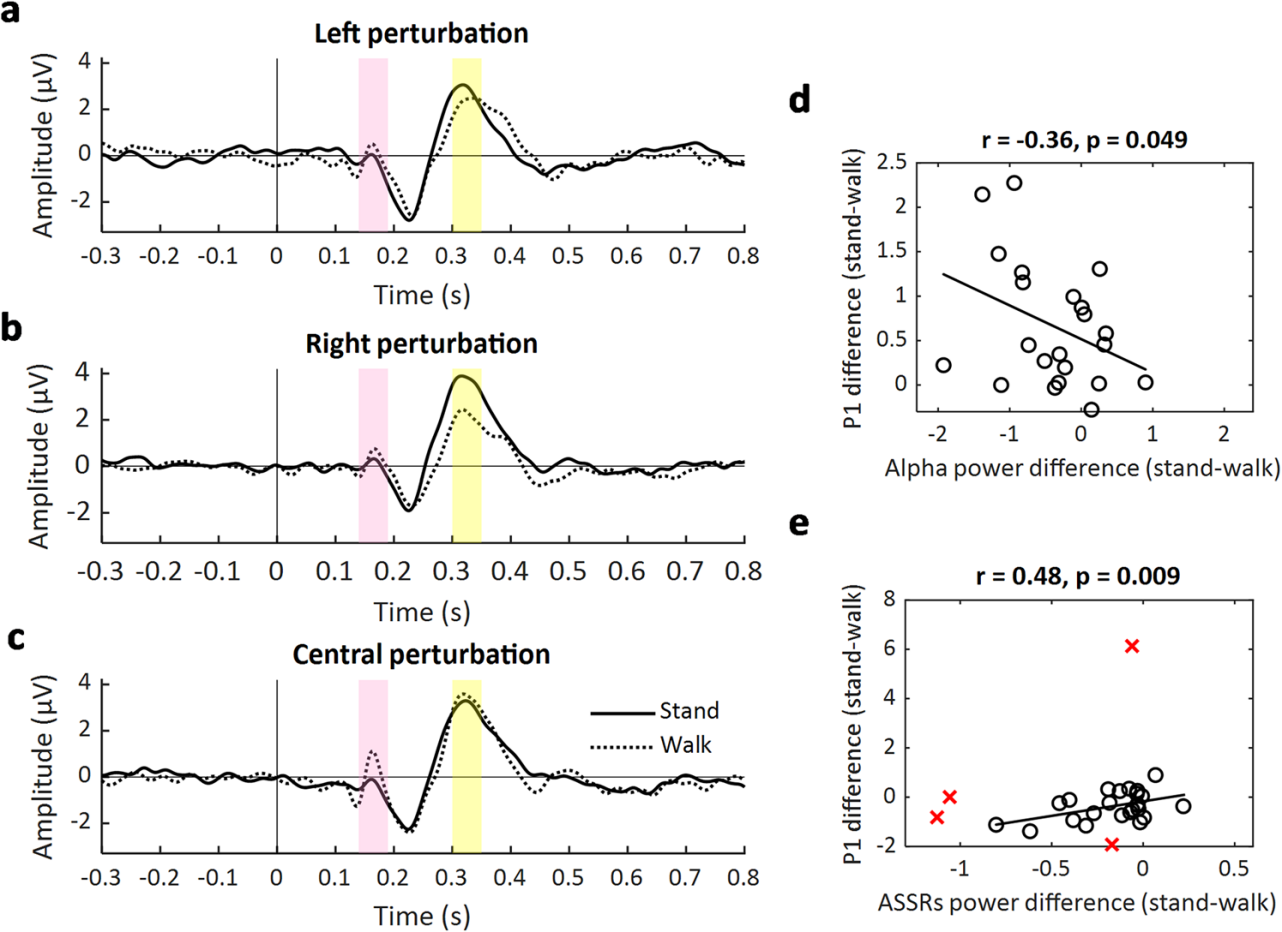
The ERP time-locked to the burst sound onset. ***a***, The ERP waveform is shown time-locked to a left burst sound during standing (solid line) and walking (dashed line). The shaded pink and yellow boxes represent the time window of P1 ([150 200] ms) and P2 ([300 350] ms) component. ***b***, Same as *** a ***but for a right burst sound. ***c***, Same as ***a*** but for a central burst sound. ***d***, The modulation of the P1 amplitude (stand–walk) negatively correlated with the modulation of ongoing alpha power (stand–walk). ***e***, The modulation of the P1 amplitude (stand–walk) positively correlated with the modulation of the ASSR power (average between 39 and 41 Hz; stand–walk).

First, a repeated-measures ANOVA with a two-way design (movement state, stand vs walk; perturbation location, left vs right vs central) was performed, revealing a significant main effect of movement state (*F*_(1,27)_ = 4.66; *p* = 0.040). The P1 amplitude was higher during walking (*M* = 0.09; SD = 0.70) compared with the standing condition (*M* = −0.27; SD = 0.09; Fig. 7*a–c*, marked in green). No other effect reached statistical significance. We further investigated the relationship between the P1 amplitude with ongoing alpha oscillations and ASSR power. The modulation of the P1 component amplitude was negatively correlated with the modulation of ongoing alpha power (stand–walk; *r* = −0.36; *p* = 0.049; one-tailed; Fig. 7*d*). This indicated that lower ongoing alpha power was associated with a higher P1 component. Furthermore, the modulation of the P1 component amplitude was positively correlated with the modulation of ASSR power (*r* = 0.48; *p* = 0.009; one-tailed; Fig. 7*e*).

We also analyzed the P2 component ([300 350] ms) using the same two-way repeated–measures ANOVA. We only observed a significant interaction between movement state and perturbation location (*F*_(2,54)_ = 4.59; *p* = 0.017), which indicates that the P2 component amplitude was smaller during walking when tone was presented dichotically (average between left and right perturbation, *t*_(27)_ = 2.70; *p* = 0.012) but showed no significant difference in the central locations (*t*_(27)_ = −0.82; *p* = 0.419).

## Discussion

### Enhanced sensory processing during natural walking

In the current study, we found that walking led to enhanced ASSR power compared with standing. The ASSR is an electrophysiological measure of brain activity that reflects the entrainment of neural activity to a rhythmic auditory stimulus (Galambos et al., 1981; Picton et al., 2002, 2009). The cortical sources of the ASSR lie within the primary auditory cortex (Herdman et al., 2003; Ross et al., 2005; Teale et al., 2008). ASSR reduction has been utilized as an indicator of clinical sensory processing dysfunction (Picton et al., 2005; Sugiyama et al., 2021). Increased ASSR power during walking suggests enhanced sensory processing in the early auditory cortex. This auditory enhancement during walking corresponds well to the findings in the visual domain, for which a higher occipital SSVEP power (induced by ongoing rhythmic visual stimulation) was observed during cycling (Bullock et al., 2017).

Notably, the ASSR power during walking was stronger than the ASSR power during stepping in place. Both conditions involve similar motor actions and somatosensory feedback, but only walking includes spatial movement. However, spatial movement independent of motor output appears to be ineffective in enhancing sensory processing (Vaughn et al., 2021). Accordingly, we propose that natural movement, e.g., motor output that has a certain ecologically valid spatial aim, introduces the strongest changes in sensory processing. Indeed, studies providing evidence for enhanced early sensory processing due to movements tested natural body movements such as walking (Chen et al., 2022a,b) and outdoor cycling (Malcolm et al., 2018; Vaughn et al., 2021). This might also explain some statistically nonsignificant effects on sensory processing during treadmill walking (Gramann et al., 2010; Malcolm et al., 2015; Nenna et al., 2020).

Additionally, we observed an amplified ERP component following a burst sound (∼150 ms after onset) while walking compared with standing. As the auditory P1 component usually has a latency of 50 ms (Hillyard and Anllo-Vento, 1998), we assume that P1 in our case was introduced by the burst sound offset (ASSR tone restart 100 ms after burst sound onset). An amplification of ERP components during movement is in line with previous studies in both the auditory (Scanlon et al., 2019; Vaughn et al., 2021) and visual (Chen et al., 2022a,b) domain. Overall, the modulation of early auditory ERP components, together with the increased ASSR signal, supports the notion of enhanced processing of auditory input due to active body movements.

### Spatially specific processing during walking

We found that dichotic burst sounds, i.e., when the burst sound was delivered to only one ear, increased ASSR perturbation during walking. This suggests a walking-induced enhancement of the processing of auditory input that is perceived to appear in a peripheral location. This is in line with previous findings in the visual domain showing that sensory processing particularly in the periphery is affected by body movement in animal (Ayaz et al., 2013) and human research (Benjamin et al., 2018; Cao and Händel, 2019; Reiser et al., 2022). Notably, the increased ASSR perturbation was not merely a side effect of elevated ASSR power during walking, as this would have also led to a walking-induced effect of the central perturbation.

Also the ERP analysis showed a spatially specific effect: the P2 component (∼200 ms after burst sound offset; ∼ 300 ms after onset) was smaller during walking than standing for burst sounds perceived to appear in the periphery (dichotic). The contrast to the walking-induced increase in ASSR perturbation suggests different underlying neural processes. However, the timing of the component does not allow us to conclusively attribute it to the onset, offset, or a blend of both stimulus events. Should it follow the offset by ∼200 ms, it may correspond to the P2 component which is associated with inhibitory processes (García-Larrea et al., 1992; Senderecka et al., 2012a,b), with stronger inhibition leading to a stronger P2 amplitude (Melara et al., 2002). In line with visual findings (Chen et al., 2022b), the observed walking-related reduction in peripheral P2 may reflect reduced inhibition of peripheral auditory input during walking.

Peripheral visual input is essential during walking to interpret motion speed and direction from the visual flow field (Banton et al., 2005; Turano et al., 2005). The here reported auditory findings suggest that the importance of peripheral input processing extends beyond vision. The auditory neural modulation might be part of a cross-modality enhancement that contributes to the perception of object movement in the surrounding environment (Rogers et al., 2017) which could ultimately assist complex processes like navigation.

### Enhanced sensory processing related to alpha power

In both experiments, we replicated the well-established reduction in alpha power due to body movements, as reviewed in the introduction. Importantly, we find that the decreased alpha power due to walking was negatively correlated with the ASSR power and P1 component amplitude. A concurrent decrease in alpha power and increase in ERPs has been reported for the auditory N1 component during outside cycling Scanlon et al. (2019) and the auditory mismatch negativity component during riding a motorcycle (Vaughn et al., 2021). Work by Cao and Händel (2019) showed a comodulation of overall alpha power and centrally visually entrained signals in different movement states. Only recently, a correlation between the visual N1 component and occipital alpha power modulation due to body movement was confirmed (Chen et al., 2022b). Based on this finding, we had proposed that, as occipital alpha power likely marks an inhibitory process, inhibition may serve as a mechanism through which walking influences early sensory processing in visual tasks (Chen et al., 2022b). The work at hand supports and extends this idea to the auditory domain.

### The strength of sensory entrainment is modulated by the walking path

By comparing the ASSR lateralization index, which reflects the difference in the entrainment strength between left and right ear, a distinct pattern was observed between turns toward left and right. The pattern showed that the entrainment strength was dynamically modulated by the turning direction (Fig. 4*a*). Before the midturn was reached, auditory processing was stronger at the side of the turn (e.g., a left turn increased processing of left ear input) suggesting a stronger processing toward the side of the turn. At midturn, the entrainment was balanced between left and right, suggesting there was no processing difference between the left and right side with respect to the participant. After midturn, processing was stronger at the opposite side of the turn.

The active sensing framework proposes that the motor system can influence perception by directly generating sensory input through the execution of motor actions (Schroeder et al., 2010; Yang et al., 2018). In human walking and other natural behavior, a number of studies has described coordinated head and eye movements to gather visual input dependent on the features of the surroundings and the specific geographic information related to the task (Imai et al., 2001; Sprague and Ballard, 2003; Turano et al., 2003; Hayhoe and Ballard, 2005). In such a way, active sensing shapes the specific content of sensory information flowing from the bottom–up (Morillon et al., 2015).

However, the current experiments delivered auditory stimuli via inserted earphones (surpassing the threshold for perceiving other external auditory input), ensuring that the auditory input remained unchanged regardless of the participants' head position. Accordingly, even so we can assume a path-related modulation of head movements, it was not the resulting change in auditory input that caused the path-specific neural response modulation. We therefore propose a top–down mechanism to modulate sensory processing during walking. The predictive coding theory suggests that the brain creates predictions about sensory input based on past experiences and current goals and that it is these predictions that guide sensory sampling and processing (Millidge et al., 2021). A review of top–down control in the active sensing framework, particularly with respect to rhythmic sampling, can be found in the work by Morillon et al. (2015).

In our experiments, the top–down modulation of sensory processing might be realized via attentional processes. These attentional processes could potentially share common neural circuitry with the systems governing eye and head movements. Previous studies showed that focusing attention to a particular side led to enhanced ASSR power in the corresponding side (Ross et al., 2004; Skosnik et al., 2007; Bharadwaj et al., 2014; Voicikas et al., 2016; Manting et al., 2020). Accordingly, the modulation of ASSR lateralization observed in our studies might reflect a shift of attention dependent on the walking path. This interpretation suggests that active sensing goes beyond an active orchestrating of the sensors and additionally includes a shift in attentional processes. Such behavior might serve to optimize navigation during natural walking. Our findings further suggest that such attentional modulation due to walking extends to the auditory domain.
